# Genome Analysis and Therapeutic Evaluation of a Novel Lytic Bacteriophage of *Salmonella* Typhimurium: Suggestive of a New Genus in the Subfamily *Vequintavirinae*

**DOI:** 10.3390/v14020241

**Published:** 2022-01-25

**Authors:** Sadia Sattar, Inam Ullah, Sofia Khanum, Marc Bailie, Bushra Shamsi, Ibrar Ahmed, Tahir Abbas Shah, Sundus Javed, Aamir Ghafoor, Amna Pervaiz, Fakiha Sohail, Kaleem Imdad, Aamira Tariq, Nazish Bostan, Ijaz Ali, Eric Altermann

**Affiliations:** 1 Molecular Virology Labs, Department of Biosciences, Comsats University Islamabad, Islamabad 45550, Pakistan; inamswati09@gmail.com (I.U.); b.shamsi11@yahoo.com (B.S.); amnapervaiz0@gmail.com (A.P.); fakihasohail96@gmail.com (F.S.); nazishbostan@comsats.edu.pk (N.B.); ijaz.ali@comsats.edu.pk (I.A.); 2 AgResearch, Palmerston North 4410, New Zealand; Sofia.Khanum@agresearch.co.nz (S.K.); marc.bailie@agresearch.co.nz (M.B.); eric.altermann@agresearch.co.nz (E.A.); 3 Alpha Genomics Private Limited, Islamabad 45710, Pakistan; alphagenomics.co@gmail.com; 4 Functional Genomics Lab, Department of Biosciences, Comsats University Islamabad, Islamabad 45550, Pakistan; syedtahirabbas@comsats.edu.pk; 5 Microbiology and Immunology Lab, Department of Biosciences, Comsats University Islamabad, Islamabad 45550, Pakistan; sundus.javed@comsats.edu.pk (S.J.); Kaleem.imdad@comsats.edu.pk (K.I.); aamira_tariq@comsats.edu.pk (A.T.); 6 University Diagnostic Lab, The University of Veterinary and Animal Sciences (UVAS), Lahore 54000, Pakistan; aamir.ghafoor@uvas.edu.pk; 7 Riddet Institute, Massey University, Palmerston North 4442, New Zealand

**Keywords:** lytic phages, *Vequintavirinae*, genome analysis, *Salmonella* Typhimurium, *Salmonella*-specific bacteriophage

## Abstract

*Salmonella* Typhimurium, a foodborne pathogen, is a major concern for food safety. Its MDR serovars of animal origin pose a serious threat to the human population. Phage therapy can be an alternative for the treatment of such MDR *Salmonella* serovars. In this study, we report on detailed genome analyses of a novel *Salmonella* phage (*Salmonella*-Phage-SSBI34) and evaluate its therapeutic potential. The phage was evaluated for latent time, burst size, host range, and bacterial growth reduction in liquid cultures. The phage stability was examined at various pH levels and temperatures. The genome analysis (141.095 Kb) indicated that its nucleotide sequence is novel, as it exhibited only 1–7% DNA coverage. The phage genome features 44% GC content, and 234 putative open reading frames were predicted. The genome was predicted to encode for 28 structural proteins and 40 enzymes related to nucleotide metabolism, DNA modification, and protein synthesis. Further, the genome features 11 tRNA genes for 10 different amino acids, indicating alternate codon usage, and hosts a unique hydrolase for bacterial lysis. This study provides new insights into the subfamily *Vequintavirinae*, of which SSBI34 may represent a new genus.

## 1. Introduction

The increasing population density has exerted significant pressure on the food production industry. Large-scale poultry and livestock rearing in developing countries require the prophylactic use of antimicrobials in subtherapeutic doses to increase production with minimum risk of disease [1,2]. This practice has contributed significantly to rapidly emerging multiple-drug-resistant (MDR) serovars of foodborne pathogens such as *Salmonella*, with zoonotic potential [3]. Such MDR *Salmonella* (Typhi, Typhimurium, and Enteritidis) pose a continuous threat to human health [4,5] and are responsible for economic losses due to their dissemination in food products [6,7]. In 2010, the WHO reported an average of 153 million cases of non-typhoidal *Salmonella* infections, with 56,969 fatalities. Around 50% of these infections were caused by contaminated food products [8,9]. *Salmonella* Typhimurium stands out amongst *Salmonella* serovars due to its zoonotic potential and broad host range [10]. The majority of *Salmonella* Typhimurium outbreaks have been caused by MDR serovars [11]. Invasive Salmonellosis requires chemotherapy, while the increasing rate of resistant serovar infections has left physicians with limited choices of drugs [12]. In Pakistan, *Salmonella* Typhimurium of poultry origin has exhibited resistance to third-generation antibiotics (fluoroquinolones and cefotaxime), leaving few choices for treatment [13,14].

The pace of antibiotic resistance acquisition has overcome the development of new and novel antibiotics due to the amount of time required to find and test new compounds. Hence, alternative strategies are needed for controlling MDR *Salmonella* serovars in reservoirs. The use of bacteriophages such as natural killers of pathogenic bacteria was proposed a century ago. Since then, phage therapy has been a subject of great interest. Bacteriophages are grouped into two broad categories: lytic phages (that replicate independently of the host DNA) and lysogenic or temperate phages (that integrate their genome into the host chromosome and later switch to lytic mode). For phage therapy lytic phages are desirable. Phages can be isolated from diverse environmental niches, including the viscera of host species and wastewaters. They are most abundant in the marine environments and are highly diverse. The 2018 International Committee of Taxonomy of Viruses (ICTV) classified bacteriophages into 5 families, 26 subfamilies, and 363 genera. Despite this diversity, phages are uniquely related to each other through multiple genetic exchanges that account for their evolution. Owing to this diversity, only a small fraction of the total bacteriophage pool has been explored and exploited for therapeutic purposes [15]. A key factor in the ongoing success of phage therapy is the discovery of new lytic phages with good lysis potential [16]. Moreover, the time required to discover and characterize novel lytic phages is much shorter in comparison to finding new antibacterials. Phage therapy has been employed in controlling pathogenic bacteria to subinfectious levels against various foodborne pathogens such as *Salmonella* [17,18,19], *Campylobacter* [20,21], and *Escherichia coli* [22,23].

In this study, we report a novel lytic bacteriophage of *Salmonella* Typhimurium with therapeutic potential. The phage genome nucleotide sequence is novel compared to existing bacteriophages in the five genera of *Vequintavirinae*. However, protein homologies place it within the members of the same subfamily.

## 2. Materials and Methods

### 2.1. Bacterial Strains and Phage Isolation

The *Salmonella enterica* subspecies *enterica* serovar Typhimurium (*S*. Typhimurium) strain SE-BS17 was isolated and characterized during this study in molecular virology labs at CUI Islamabad Pakistan using standard protocols. Briefly, poultry organ samples (caecum, intestine, liver, and spleen) were collected from poultry farms in the federal area supplying meat to retail poultry shops in Rawalpindi and Islamabad. The poultry samples were collected in sterile bags. Twenty-five grams of each poultry tissue sample (all tissue types pooled together) was homogenized to a fine paste using sterile surgical blades and pre-enriched with 225 mL of 0.1% buffered peptone water (Oxoid) and incubated overnight at 37 °C. After pre-enrichment, 100 μL of broth culture was transferred to selenite cystine broth (Oxoid) followed by incubation at 37 °C for 24 h. One loopful of enriched broth was streak-plated onto xylose lysine deoxycholate (XLD agar, Oxoid; CM0469) and incubated at 37 °C for 24 h. The plates were examined for typical colonies of *Salmonella* [24]. All isolates exhibiting typical *Salmonella* colonies on the XLD plate were subcultured for biochemical characterization with API 20 E strips (bio-Merieux; Ref No. 20100) as per the manufacturer’s protocols. Isolates identified as *Salmonella* (38 out of 65) by biochemical or numeric profiling of the API kit were further subjected to PCR amplification of the 284 bp *Inv* A gene for the detection of the *Salmonella* genus [25] and *Iro* B gene (606 bp) for the detection of subspecies (Table S1). Out of these isolates, one strain, SE-BS17, was used for bacteriophage isolation on the basis of resistance profile. The strain was later characterized by 16 S rRNA gene sequencing (Macrogen, Seoul, Korea) for serovar identification (Gen Bank Acc. No; MZ503545). Further, it was tested for antibiotic sensitivity against common antibiotics used in poultry using the Kirby–Bauer disc diffusion method [26]. SE-BS17 was tested for the presence of beta-lactamase (*bla*TEM-1, 643 bp) [27], extended-spectrum beta-lactamase (*bla*CTX-M, 754 bp) [28], and the trimethoprim resistance gene *dfr*A1 (474 bp) [29] by PCR amplification. The PCR was carried out as per methods given in the publications cited above; briefly, the reaction was carried out in 40 μL of solution by adding 8 μL master mix (Solis Biodyne, Cat No. 04-11-00S15), 29 μL sterile water, 2 μL (10 pmol) of each forward and reverse primer, and 1 μL of template DNA (250 ng). Amplification was carried out in a thermocycler (Thermo Fisher, Waltham, MA, USA) under the following conditions: initial denaturation at 95 °C for 5 min; 35 cycles of denaturation at 95 °C for 30 s; different annealing temperatures as per the GC content of each primer given in respective publications. Elongation was carried out at 72 °C for 45 sec, and the final extension was performed at 72 °C for 8 min. PCR products were subjected to gel electrophoresis with a DNA ladder (Solis Biodyne, Cat No.07-12-0000S) as the molecular marker (Supplementary Table S1).

For bacteriophage isolation, 1 cm tissue fragments of the intestine, stomach, and caecum from poultry samples used for *Salmonella* isolation were triturated and inoculated in 9 mL of selenite cystine broth (Oxoid) in 15 mL Falcon tubes and incubated at 37 °C for 24 h without shaking. After incubation, 1 mL culture from each tube was centrifuged at 14,000 rpm (Centrifuge, Hermle, Siemensstr. 25, D-78564 Wehingen, Germany ZK 496) for 20 min at room temperature. The supernatant was filtered through a 0.22 μm syringe filter (CNW Technologies, Düsseldorf, Germany) and tested for the presence of phages by spot assay using the agar overlay method as described by Bao et al. [30]. Briefly, 2.5 mL of Luria–Bertani (LB) soft agar (0.5% *w*/*v*) was mixed with 250 µL of an overnight culture of SE-BS17 and poured onto solidified LB agar plates. The plates were allowed to solidify for 15 min. Next, 10 µL filtered phage lysate was placed on the soft agar plates and allowed to dry. Plates were then inverted and incubated at 37 °C for 24 h. Samples producing clear circular zones on the bacterial lawn were indicative of phage activity and were selected for further purification and characterization.

### 2.2. Single Plaque Purification

For plaque purification and amplification, the pour plate method was used as described elsewhere [31]. Briefly, 25 µL of positive phage lysate and 250 µL of SE-BS17 strain overnight culture were mixed in 2.5 mL of soft agar and poured onto solidified LB agar plates (Oxoid) (Agar overlay method). Plates giving countable plaques were selected further. One isolated plaque was pierced and resuspended in 1 mL phosphate-buffered saline (PBS, (137 mM NaCl, 2.7 mM KCl, 8 mM Na_2_HPO_4_, and 2 mM KH_2_PO) at pH 7.2, followed by gentle agitation for 1 h and subsequent centrifugation at 8000 rpm for 10 min. The supernatant was filtered through a 0.22 µm syringe filter and kept at 4 °C. Lysates were propagated in SE-BS17 and isolated twice more to obtain pure phage cultures. The phage was named *Salmonella*-phage-SSBI34 (SSBI34).

### 2.3. Large-Scale Amplification of SSBI34

An overnight culture of SE-BS17 was diluted (1:100) in 1 L LB medium and incubated at 37 °C with shaking until it reached log phase (OD_600_ 0.55) to increase the phage titer of the isolated bacteriophage SSBI34. The culture was then inoculated with phage lysate at MOI 1 and allowed to grow for 24 h. The culture was then centrifuged at 8000 rpm (Centrifuge, Hermle, Siemensstr. 25, D-78564 Wehingen, Germany ZK 496) for 10 min, then the supernatant was filtered and stored at 4 °C. Phages were concentrated via polyethylene glycol 8000 (PEG) (Sigma Aldrich Cat. No. 1546605) precipitation [32]. Briefly, PEG (10% *w*/*v*) and NaCl (2.5 M) were dissolved in phage lysate and incubated at 4 °C overnight. The next day the samples were precipitated by centrifugation at 10,000 rpm (Helmer, Germany Cat No. ZK 496) for 45 min at 4 °C. The precipitate was dissolved in 2 mL PBS (pH 7.2), filtered through a 0.22 µm syringe filter, and stored at 4 °C. The phage titer was determined by spotting and enumerating serial two-fold dilutions on agar overlay plates as described above.

### 2.4. Phage Stability and One-Step Growth Curve

SSBI34 stability was tested by incubating phage particles suspended in PBS at various temperatures and pH values [33]. Briefly, 7.23 × 10^5^ pfu/mL phage particles were incubated for 1 h in 1 mL PBS at pH 7.2 and at 37 °C, 55 °C, 70 °C, and 80 °C and then titrated. For pH stability, PBS at different pH values (pH 2, 5, 7, 9, and 12) adjusted with either 6 M HCl or 6 M NaOH was prepared and phages were diluted to a final concentration of 1.4 × 10^5^ pfu/mL. Following incubation at 37 °C for 1 h, the phages were plated as described previously. The phage burst size and latent period were determined using the one-step growth curve method as stated by Kropinski [34], with slight modifications. Briefly, SE-BS17 overnight culture was diluted (1:100-fold) in 30 mL LB medium and allowed to grow until log phase (OD_600_ 0.5) at 37 °C. Cultures were then infected with SSBI34 at MOI 1 and phages were allowed to adsorb without shaking for 20 min. The culture was then centrifuged at 10,000 rpm (Centrifuge, Helmer, Germany Cat No. ZK 496) for 10 min, the supernatant was removed, and the bacterial culture was suspended in 30 mL of fresh LB medium and allowed to grow at 37 °C. The supernatant was titrated for unabsorbed phage particles by sampling 1 mL aliquots at 5 min intervals for 70 min. Samples were centrifuged, filtered, and then titrated. The burst size of SSBI34 was calculated using the following formula:

Burst size = Phages after burst (C) − Free phages (B) = New phage released (D)

Total applied phages (A) − Free phages (B) = Number of infecting phages (E)

Details are given in Supplementary Table S2.

### 2.5. Host Range

The SSBI34 host range was determined using the agar overlay spot method [35]. Initially, a spot was placed on double-layer agar plates, then later for all strains showing lysis or clear zones or plaques, a serial two-fold dilution of phage-rich lysate was made and tested using the double-layer agar method. Bacterial strains used for host range determination included *Klebsiella pneumoniea* (OK086689), *Staphylococcus aureus* (OK086690)*,* Uro-pathogenic *Escherichia coli, Salmonella Typhi, Enterococcus facialis* (MZ496438)*, Streptococcus pyogenes*, *Pseudomonas aeruginosa, Acinetobacter baumannii* (MZ496431), *Enterobacter hormaechei* (OK086761), *Citrobacter* sp., and 14 strains of *Salmonella enterica*. Accession numbers are provided where available. Briefly, an overnight (ON) culture of each strain was grown in a 5 mL LB medium. Here, 250 µL of ON culture was mixed with 2.5 mL of soft agar (0.5% agar dissolved in LB medium), poured onto already solidified LB agar plates, and solidified for 20 min. Next, 10 µL drops of phage lysate were plated on each plate and allowed to dry for 10 min. Plates were then inverted and incubated overnight at 37 °C. The presence of clear zones was indicative of phage growth and indicator strain sensitivity.

### 2.6. Bacterial Growth Reduction Assay

The ability of SSBI34 to lyse *S.* Typhimurium (SE-BS17) was determined by bacterial growth reduction assay as described by O’Flynn [36]. An overnight culture of *S.* Typhimurium strain SE-BS17 was diluted in 40 mL of LB broth in three 100 mL flasks for each test unit to a final concentration of 1.0 × 10^5^ CFU/mL. Three flasks were infected with SSBI34 at MOI 1. Here, 40 ml LB medium inoculated with 1.0 × 10^5^ CFU/mL bacterial culture was used as a control. All flasks were kept at 37 °C and allowed to grow for 24 h. Every 2 h, a 1 mL aliquot was removed from all flasks and the optical density was measured at 600 nm using a spectrophotometer (Cat No. 721-100G, Hinotek, Ningbo, China). Values were plotted in the graph as the averages of triplicates and statistical significance is given where required. A standard curve was plotted to determine bacterial colony-forming units at various optical density values using a dilution plating technique. This curve was used to determine the CFU/mL at the corresponding OD600 nm level for the growth reduction assay.

### 2.7. Transmission Electron Microscopy

In total, 5 µL of PEG purified phage lysate (~7 × 10^8^ pfu/mL) suspended in distilled water was placed on glow-discharged, carbon-coated grids and incubated at room temperature for 5 min. Later, the grids were washed with 5 µL of ddH_2_O and incubated with uranyl acetate solution (2%) for 2 min. Electron microscopy was carried out at MMIC (Manawatu Microscopy and Imaging Centre), Massey University, Palmerston North, New Zealand.

### 2.8. Genome Sequencing and Bioinformatic Analysis

SSBI34 genome was sequenced using the Illumina MiSeq platform at Massey Genome Service (Massey University, Palmerston North, New Zealand). Briefly, the phage DNA library was prepared using Nextera^TM^ XT library kit_V2 (Illumina, San Diego, CA, USA). The phage genome was digested by enzymes into random fragments. Sequencing created 301,237 pair-end (PE) reads with an average read length of 2 × 151 bps. The software was run using default parameters unless otherwise specified. Quality control was performed using FastQC (version 0.11.3) before and after trimming with Trimmomatic (Version 0.39). The genome was assembled with plasmidSPAdes (version 3.13.2). The assembly graph was inspected using Bandage (v0.8.1). The assembly quality was assessed using QUAST (version 5.0) and SQUAT (Dec 2019). The genome was automatically annotated using the GAMOLA2 software package.

### 2.9. Phylogenetic Analysis of SSBI34

Whole-genomic sequences of 12 viral genomes having BLASTn homology in NCBI were used for the phylogenetic analyses using the online resource VICTOR (https://ggdc.dsmz.de/victor.php, accessed on 4 January 2022). All pairwise comparisons of the nucleotide sequences were conducted using the genome BLAST distance phylogeny (GBDP) method [37] under settings recommended for prokaryotic viruses [38].

The resulting intergenomic distances were used to infer a balanced minimum evolution tree with branch support via FASTME, including SPR postprocessing [39] for the D0 formula. Branch support was inferred from 100 pseudo-bootstrap replicates each. Trees were rooted at the midpoint [40] and visualized with FigTree [41]. Taxon boundaries at the species, genus, and family levels were estimated with the OPTSIL program, the recommended clustering thresholds, and an F value (fraction of links required for cluster fusion) of 0.5.

Capsid protein gene sequences from these genomes were extracted and aligned together in Geneious (v.8.1.9) [42]. Alignments were trimmed from both ends and the gapped sequences or insertions and deletions were removed from the alignment. The alignments in Phylip format were imported in IQtree [43] online version [44] available at http://iqtree.cibiv.univie.ac.at/ (accessed on 10 November 2021) to reconstruct a maximum likelihood tree based on the best fit model as implemented in the IQTree, using a bootstrap value of 1000. IQTree implements ModelFinder [45] to calculate the best fit model for the data. The trees in Newick format were refined in the online TreeDyn tool [46] available at http://www.phylogeny.fr/one_task.cgi?task_type=treedyn (accessed on 10 November 2021) and downloaded in pdf format.

### 2.10. Statistical Analysis

Statistical analyses were performed using Origin 2019 (Origin Lab, Northampton, MA, USA). The statistical significance was determined using ANOVA followed by Tukey’s honest significant difference (HSD) test for specific comparisons. Statistical significance was reached at *p* < 0.05.

## 3. Results

### 3.1. Phage Isolation and Characterization

**Figure 1:**
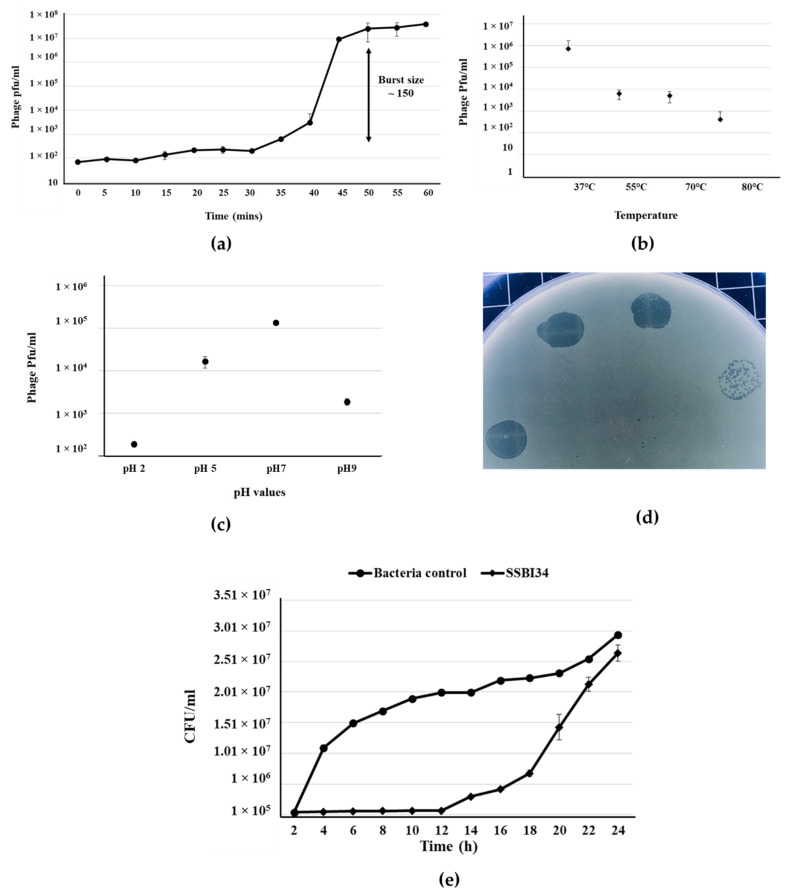
*Salmonella*-phage-SSBI34 characterization. (**a**) One-step growth curve of SSBI34 when grown on SE-BS17 (MZ503545) at 37 °C in LB medium, with the burst size calculated as per the formula given in the Materials and Methods. Stability of SSBI34 (**b**) when exposed to various temperatures (37 °C–80 °C) and (**c**) when exposed to various pH values (pH 2–pH 9). (**d**) Phage plaques on double-layer agar plates. (**e**) Bacterial growth reduction assay. SSBI34 was able to maintain the bacterial population to background levels for 12–16 h. All values are shown as means ± standard error.

*Salmonella*-phage-SSBI34 was isolated from retail poultry samples using *S.* Typhimurium (SE-BS17) (Gene Bank Acc No. MZ503545) as the host strain. SE-BS17 is a multi-drug-resistant strain of *S.* Typhimurium having resistance against tetracycline (TE), kanamycin (K), sulphamethoxazole-trimethoprim (SXT), erythromycin (E), ampicillin (AMP), neomycin (N), novobiocin (NV), streptomycin (S), and nalidixic acid (NA). Here, it was also positive for beta-lactamase (*bla*TEM-1) and extended-spectrum beta-lactamase (*bla*CTX-M) genes. The host adsorption efficacy of SSBI34 was 98% within 10 min of adding SSBI34 to log-phase bacterial culture. The phages’ latent period as determined by one-step growth curve was 35 min, with a burst size of ~150 pfu/cell (Figure 1a). The phage produced a maximum titer at 37 °C; however, it was stable at high temperatures as well. Temperature analysis indicated that SSBI34 was still infective after incubation at 55 °C, 70 °C, and 80 °C, albeit with a significant drop in titer. Beyond 80 °C, it lost its infectivity, for example 100 °C it produced no plaques (Figure 1b). SSBI34 tolerated pH extremes very well. The phage was stable and infective at pH 2, 5, 7, and 9, despite a significant drop in titer (Figure 1c).

Phage had a good lysis potential, as observed by the high titer (1 × 10^8^ pfu/mL) and clear zones of lysis on agar plates (Figure 1d). SSBI34 is a lytic phage, and it restricted the growth of *S.* Typhimurium for 12 h at background levels, after which phage-resistant mutants started appearing. The growth of *S.* Typhimurium increased after 12 h; however, it was significantly lower than the bacteria-only control for another 4 h (Figure 1e) (*p* < 0.05). The SSBI34 host range was tested on 14 *Salmonella* strains. These strains were characterized up to the subspecies level (materials and methods) and all belonged to *Salmonella enterica* subspecies *enterica*; however, their serovars were not known. SSBI34 produced clear zones of lysis on two strains of *S.* Typhimurium only. Few other species of the family *Enterobacteriaceae* tested by spot method were not lysed. It is difficult to conclude the definitive host range of SSBI34, as fewer strains were available for testing.

**Figure 2:**
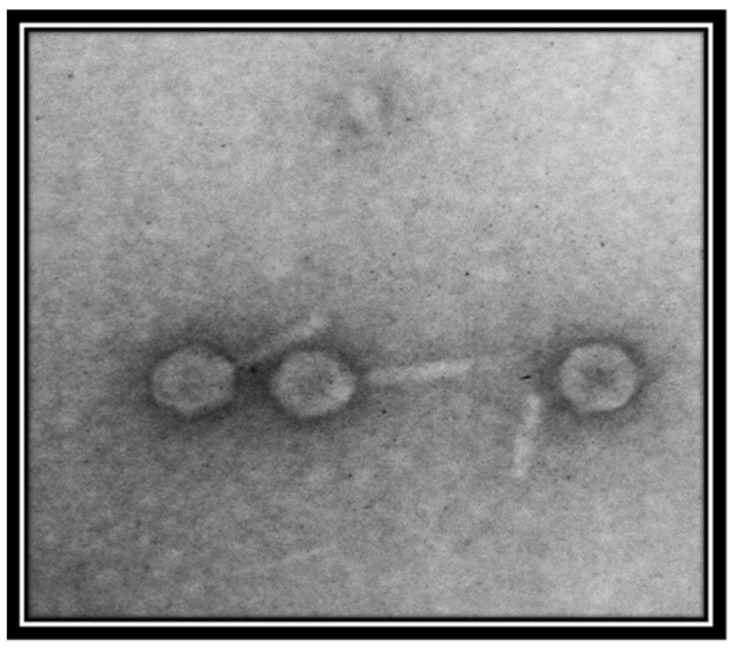
Transmission electron micrograph of *Salmonella*-phage-SSBI34. The phage was stained with 1% uranyl acetate solution. Images were taken at an acceleration voltage of 80 kV. The scale bar represents 100 nm.

Transmission electron micrography showed that the phage is a member of the family *Myoviridae*, as indicated by the presence of a clearly identifiable hexagonal head and tail (Figure 2). However, the resolution of the image was not good. For further evaluation, better quality images are required.

### 3.2. Genome Characterization of Salmonella-Phage-SSBI34

Genome sequencing of SSBI34 indicated a double-stranded DNA genome of 141.095 base pairs (coverage~500×) with a GC content of 44%. SSBI34 did not show any significant nucleotide homology when compared with general nucleotide sequences in BLASTn. The maximum nucleotide homology was observed with the *Klebsiella* phage vB_KaeM_KaOmega (7%), *Cronobacter* phage CR9 (5%), and *Cronobacter* phage CR3 (4%). This nucleotide homology was observed only for three regions in genome-encoding putative major capsid protein (79.41%, *Cronobacter* phage CR9), putative DNA primase/helicase (77.25%, *Pectobacterium* phage DU_PP_I), and a conserved hypothetical protein with members of the genus certrevirus (87.91%; *Pectobacterium* phage phiTE and *Cronobacter* phage CR3; 94.49%). In the absence of any significant nucleotide homology, amino acid sequencing of putative ORFs was individually compared in BLASTp against the non-redundant protein database.

**Figure 3:**
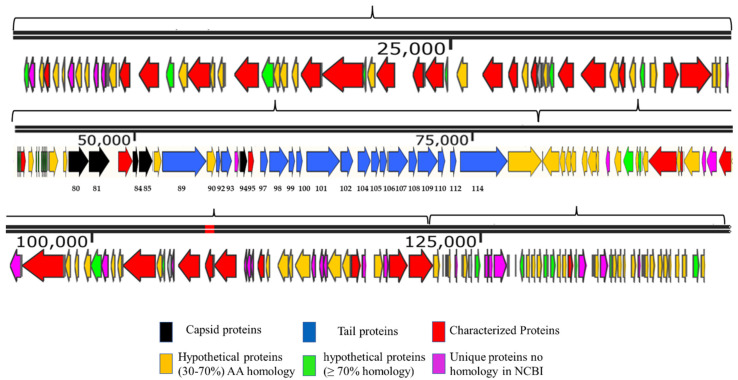
Genome map of SSBI34 (141.095 Kb). The directions of arrows represent the replication strands (+ or −). Different ORFs are color-coded to reflect their characteristics. ORFs having BLASTp homology with characterized phage proteins in NCBI are colored red. Black ORFs are involved in the capsid head formation, blue ORFs are involved in tail formation, orange ORFs are hypothetical proteins having BLASTp homology of between 30 and 70%, whereas Green ORFs are hypothetical proteins with BLASTp homology of between 70 and 90%. Purple ORFs have no significant homology with any protein in NCBI or BLASTp and are unique to SSBI34. The upper line represents the scale bar. Numbers at the bases of ORFs represent ORF numbers corresponding to structural proteins given in Table 2, whereas all other protein homologs are given in Supplementary Table S2.

**Figure 4:**
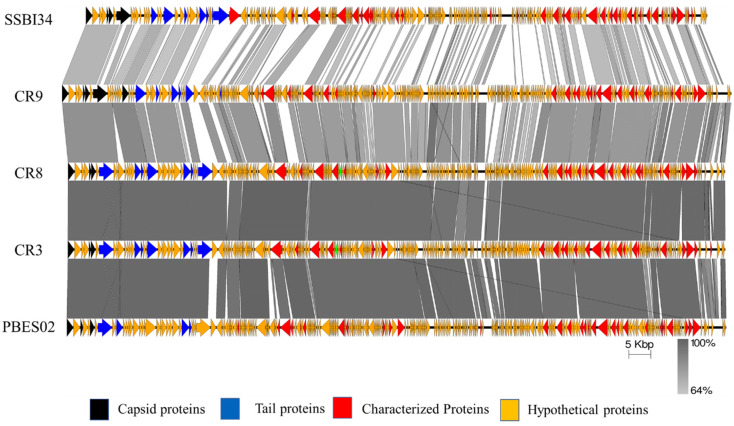
Easyfig homology diagram of SSBI34 with close phage relatives in NCBI using BLASTp. Various ORFs are color coded according to their putative function (scheme provided at the base of the figure).

The genome map of SSBI34 is given in Figure 3. All open reading frames (ORFs) are color-coded according to their homology and putative function. The genome has 234 ORFs; however, only 44 (19%) ORFs have ≥75% amino acid sequence homology with any protein in the NCBI database (Table 1 and Table S2). Out of a total of 234, only 61 ORFs have similarities (30–90%) with the characterized protein sequence in the NCBI database (BLASTp). The remaining 173 ORFs were all hypothetical proteins with no defined function. Hypothetical proteins were divided into three categories based on amino acid homology in BLASTp: (1) hypothetical proteins unique to SSBI34, having no significant homology with any protein in the database (Figure 3, purple ORFs, 15.6%); (2) hypothetical proteins having 30–70% amino acid homology with different proteins, mostly with members of *Vequintavirinae* (orange, 44%); (3) hypothetical proteins having ≥70% homology with different proteins in BLASTp (green ORFs, 12.7%). The majority of SSBI34 proteins showed homology to various members of the subfamily *Vequintavirinae*. However, the homology was not significant enough to any member to classify SSBI34 into any of the five genera of *Vequintavirinae* (see Discussion section). A linear comparison figure of multiple loci of SSBI34 with close phage homologs was generated using Easyfig software (Figure 4).

#### 3.2.1. Structural ORF Analysis of SSBI34

Overall, *Cronobacter* phages CR9 and CR3 contained the highest structural protein similarity with SSBI34 at 17.52% and 15%, respectively. For ease of illustration, structural proteins having BLASTp homology were divided into two categories and are color-coded (Figure 3, Table 1): (1) those involved in capsid formation and DNA packaging into the already made prohead (black color); (2) those involved in tail formation (blue color). In total, five proteins of SSBI34 showed homology with capsid structural proteins of CR9 and CR3 (*Vequintavirinae*, genus *certrevirus*), including PBSX family large terminase (packaging of DNA, CR3), putative portal protein (CR9), putative prohead protease (CR9), putative head stabilization/decoration protein (CR9), and putative major capsid protein (CR9). In the second category, 19 proteins were involved in tail formation with varying degrees of homology in BLASTp (Table 1, Figure 3). Contrary to capsid proteins, tail proteins exhibited similarity to proteins of different members of various genera in *Vequintavirinae*. Out of 19 proteins associated with tail formation, those involved in tail fiber production and assembly, namely tail-to-head connector protein, putative tail tube, base plate wedge protein, and tail assembly chaperon protein, all had high homology with *Cronobacter* phages CR9, CR3, and CR8. Majority putative tail fiber proteins possess the collagen domain considered a hallmark for phage proteins involved in tail fiber formation. Proteins involved in tail tape measure, base plate assembly, and the base plate wedge lysozyme had homology with *Pectobacterium* phage DU_PP_I, *Escherichia* phage UPEC06, *Cronobacter* phage PBES02, and *Klebsiella* phage vB_KaeM_KaOmega, respectively.

#### 3.2.2. ORFs Involved in RNA Synthesis and Modification

The SSBI34 genome possesses 11 tRNA genes for 9 amino acids—two for methionine and one each for threonine, asparagine, aspartate, isoleucine, proline, glycine, and serine. It also has two tRNA genes for selenocysteine, the 21st proteinogenic amino acid. In addition to selenocysteine tRNA, the phage also contains serine tRNA ligase. Serine tRNA ligase is an enzyme required to load selenocysteine to its tRNA in a specific two-step mechanism. It allows the phage to use an internal stop codon. SSBI34 possesses four enzymes for various modifications of RNA. ORF 63 (Supplementary Table S2) encodes queuosine biosynthesis protein QueD, whose product queuosine is a hyper-modified base involved in wobble pairing of tRNA, and it increases the efficiency and rate of mRNA production in lytic life cycles. It also has an RNA polymerase (Table S2, ORF 95), indicating partial independence from host RNA protein synthesis machinery. An RNA triphosphatase catalyzes the first step in adding 5′ cap to newly synthesized mRNA (ORF 134), an RNA ligase of Rnl2 family involved in splicing editing and repair of RNA (ORF166), as well as a serine tRNA ligase involved in appropriate attachment of amino acids to tRNA molecules (ORF 17). It also has an ORF coding for ribosomal protein uS19, rpsS, with a possible role in translation initiation (ORF 191).

#### 3.2.3. DNA Replication, Modification, and Metabolism

The genome analysis of SSBI34 indicated the presence of several enzymes associated with DNA bases metabolism, modification, and replication. Supplementary Table S2 summarizes the details of these ORFs. For DNA synthesis, SSBI34 has two DNA polymerases, DNA pol III and DNA pol I (ORF 45 and 142), indicating SSBI34′s independence from host polymerases for DNA replication. It also encodes a DNA ligase (ORF 61) and a replicative phage helicase involved in unwinding DNA for replication (ORF 154). These two enzymes provide double-strand nick repair via non-homologous recombination. SSBI34 also contains a Ti-type conjugative transfer relaxase *TraA*, a single-strand exonuclease involved in unwinding DNA before replication (ORF 162). A homolog of endonuclease VII of T4 phages was also present for ease of packaging and mismatch repair (ORF 47). Genome housed several enzymes for nucleotide metabolism, such as ATP-dependent Clp endopeptidase (ORF 29), tRNA nucleotidyltransferase (ORF 19 and 24), ribonucleoside-diphosphate reductases (conversion of nucleotides to deoxynucleotides) (ORF 37,38), exodeoxyribonuclease (nuclease, ORF 51), and glutaredoxin 3 involved in DNA synthesis (ORF 34). In addition, two HNH endonucleases (ORFs 52 and 75) involved in DNA packaging into already made prohead were detected in the SSBI34 genome. Phage SSBI34 possesses an ORF (41) coding thymidylate synthase that is flavin-dependent (thyX). This enzyme is involved in the de novo synthesis of thymidine from flavin rather than the usual pathway and enables phage replication in folate-deficient environments.

SSBI34 also has four ORFs involved in host and phage DNA modifications leading to phage DNA protection and ease of replication. An anti-restriction endonuclease (ORF 55) capable of modifying phage bases to escape the bacterial restriction modification (R-M) system can also be found (Supplementary Table S2). This enzyme changes the recognition site of bacterial R-M (restriction modification) enzymes in the phage genome, thereby protecting the phage genome from hydrolysis. Another putative protein involved in phage DNA protection is the restriction alleviation protein Ral. It modulates the host R-M system and restricts the cleavage of phage DNA (ORF 146) [47]. Together these two proteins may function to trick the host restriction modification system to protect phage DNA, allowing better replication and phage survival. After DNA synthesis, two other enzymes are involved in phage DNA methylation. Putative DNA-[N6-adenine] methyltransferase of T4 phages catalyzes the methylation of adenine residues at the N6 position. The second protein with similarity to the methanogen marker protein is also present, which may be involved in phage production under anaerobic conditions (ORFs 1 and 28).

#### 3.2.4. Cell Wall Hydrolases

**Figure 5:**
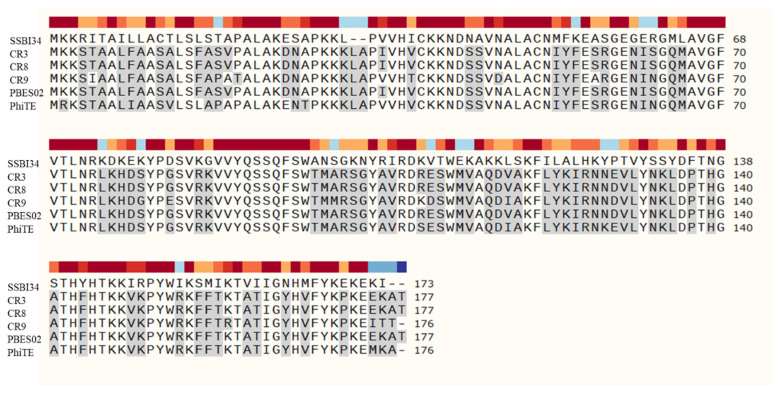
Amino acid sequence comparison of hydrolases in *Vequintavirinae* using SnapGene software version 5.3. SSBI34 has no nucleotide homology in BLASTn, whereas only 50% amino acid sequence homology was observed with other hydrolases. The hydrolases from other members of *Vequintavirinae* show high homology with each other. All amino acid residues in other phages that are different from SSBI34 amino acid residues are highlighted in grey.

Two putative cell wall hydrolases were detected in the SSBI34 genome. ORF106 (Table 1) encoded an acidic lysozyme as part of the base plate wedge protein in SSBI34. This protein is thought to be involved in the attachment and lysis of peptidoglycan at the time of phage entry. The second cell wall hydrolase identified in SSBI34 was a homologue of SelB, a spore cortex lytic enzyme of *Bacillus subtilis*. ORF 165 in the SSBI34 genome encoded a 173 AA hydrolase having 50.2% homology with spore cortex lytic enzymes of *B. subtilis*, an effective peptidoglycan hydrolase involved during spore germination. It is presumed that this enzyme may be responsible for peptidoglycan hydrolysis and phage release (Figure 5).

#### 3.2.5. Phylogenetic Analysis of SSBI34

Phylogenomic GBDP trees were inferred using the whole-genome sequence of the 12 closest homologs of SSBI34 using the D0 formulas and yielding average support of 30%. The numbers above branches are GBDP pseudo-bootstrap support values from 100 replications. The branch lengths of the resulting VICTOR trees are scaled in terms of the respective distance formula used. The OPTSIL clustering for the D0 yielded eleven species clusters, one genus cluster, and one family cluster. In this tree, the *Acinetobacter* phage ABPH49 is the outermost outlier. The *Salmonella* phage SSB134 formed a second outlier group with the *Escherichia* phage UPEC06. The rest of the taxa formed one cluster with low iteral bootstrap support values.

**Figure 6:**
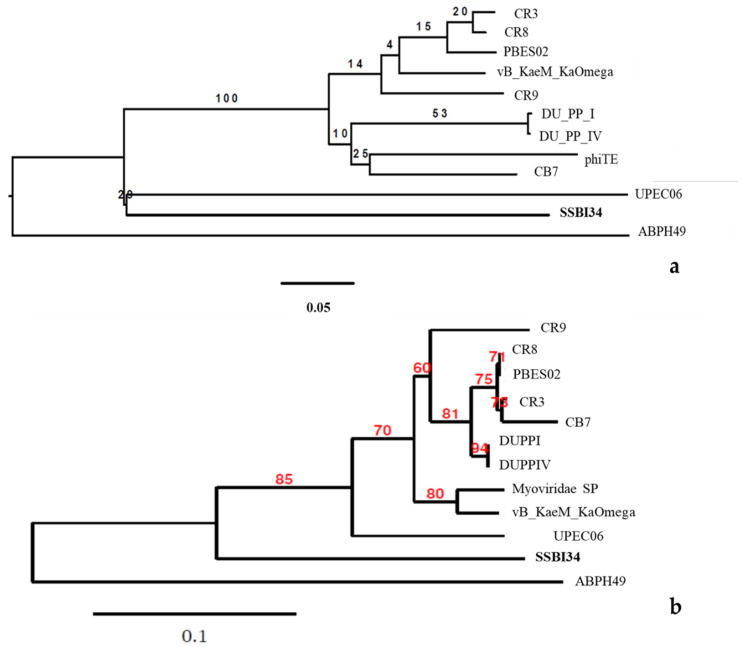
(**a**) The BLAST distance tree using whole-genome sequence of 12 homologs in BLSTn provides a rough estimate of the relationships between related sequences in NCBI and *Salmonella*-phage-SSBI34. (**b**) Phylogenetic tree using the capsid protein gene of these closest homologs.

For comparison purposes, the capsid gene sequence of the abovementioned 12 phages was used to build the phylogenetic tree. The modified alignment after removing the gaps was 842 nucleotides long. The best fit model for the data was found to be K3P + R2. CR8 and PBES02 capsid genes were identical, while DUPPI and DUPPIV had identical capsid gene sequences. The basal group was ABPH49. The maximum likelihood tree produced by the TreeDyn program is given in Figure 6b. The tree is unrooted indicating the absence of most recent common ancestor (MRCA). The tree shows the P-distances. In this tree, ABPH49 is an outlier, while the rest of the taxa arrange themselves in the form of a single cluster. At the secondary node level, our phage SSB134 showed significant divergence from the rest of the cluster’s sister taxa, with an 85% boot strap support value, while the rest of taxa showed variable bootstrap values at tertiary and quaternary node levels.

## 4. Discussion

**Figure 7:**
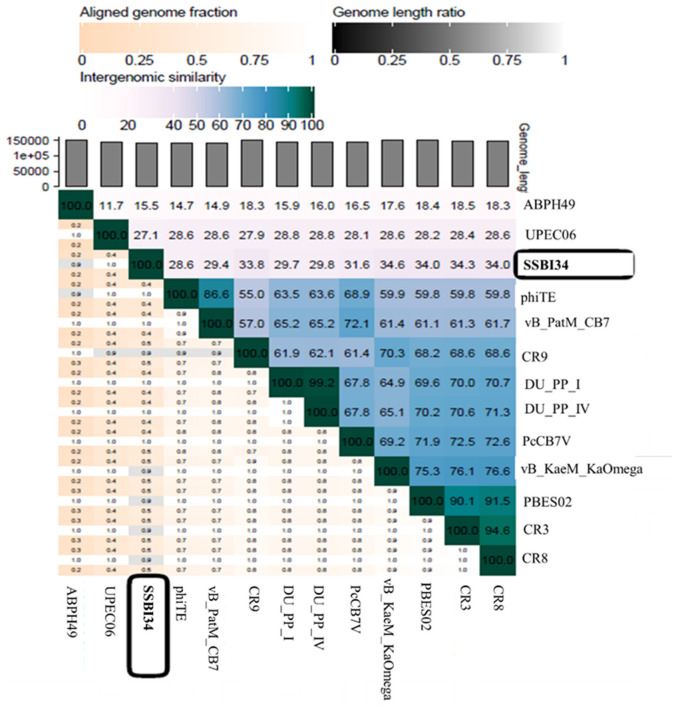
Heatmap generated after comparative genome analysis of *Salmonella*-phage-SSBI34 with its close homologs in BLASTn using VIRIDIC software.

According to the recent classification of ICTV released in March 2020, the family *Myoviridae* is divided into eight subfamilies, 217 genera, and 625 species. One subfamily, *Vequintavirinae*, is further subdivided into six genera named *Avunavirus*, *Certrevirus*, *Henunavirus*, *Mydovirus*, *Seunavirus*, and *Vequintavirus*. In addition, a division with no rank assigned is called the “unclassified *Vequintavirinae*”, which includes eight viruses awaiting classification [48]. According to ICTV, any new virus that is isolated and sequenced can be classified into a particular genus based on one of two parameters: (1) viruses with >50% nucleotide homology with members of a particular genus; [49] (2) viruses with 40% of their virus having ≥75% homology with the members of that particular genus [50]. When we analyzed *Salmonella*-phage-SSBI34 (SSBI34) according to this classification criterion, we found that it does not fulfill either criterion. Only 7% of the genomes of SSBI34 had homology (77.94%) with any virus in the database. The nucleotide sequence was unique and was not similar to any virus stored in the NCBI database. A heat map was generated using VIRIDIC software for comparative genome analysis of SSBI34 with its close homologs. This indicated its unique nature (Figure 7). Moreover, SSBI34 has 234 putative ORFs, out of which only 52 ORFs (22.2%) had ≥75% homology with various members of the different *Vequintavirinae* genera, as indicated by BLASTp in NCBI. So far, the subfamily *Vequintavirinae* (family Myoviridae) has only two *Salmonella* phages in the order *Seunavirus* (NCBI Acc. NO. NC_016071.1, NC_027351), while *Salmonella*-phage-SSBI34 is entirely different from both (Figure 7).

If we compare the protein homology of SSBI34 with individual *Vequintavirinae* genera, this indicates that overall 70% of proteins of SSBI34 have 30–90% BLASTp homology with different members (CR3, CR9, CR8 and, PBES02; Figure 4) of the *Certrevirus* genus; however, out of these proteins, only 17% of ORFs have more than ≥75% BLASTp homology. Moreover, SSBI34 also had low protein homology (9.2% overall, ≥75% = 1.3%) with two viruses *Acinetobacter* phage ABPH49 and *Escherichia* phage UPEC06, indicating that the two criteria required to place SSBI34 in the existing genera were not fulfilled. We suggest the creation of a new genus in the subfamily *Vequintavirinae* for phage SSBI34. Since ICTV recommends at least two characterized genomes to create a new genus, until then SSBI34 can be placed with unclassified *Vequintavirinae* phages. The same was evident from the phylogenetic analysis of SSBI34, which places it as a separate lineage from other genera of *Vequintavirinae* (Figure 6a,b).

Three major phages, CR3, CR8, and CR9, whose proteins are closely related to SSBI34, have not been physically characterized to date, and only their genomes have been reported [51,52,53]. In the absence of any close relatives, very few can be compared with other members of *Vequintavirinae*. SSBI34 has a genome size of 141.095 Kb, which is well within alignment with other viruses of *Vequintavirinae*, whose genomes fall between 141 and 151 Kb (Table 2) [53]. The GC content of SSBI34 was 44%, lower than most phages having homology with SSBI34 (50% or higher) and the host *S.* Typhimurium. The burst size of SSBI34 was less than that reported for other phages of the *Certrevirus* genus (250 pfu/mL). However, the burst time was comparable to CR3 and PBES02 (30 min) [54]. SSBI34 was more tolerant to pH and temperature extremes than other phages of *Vequintavirinae*, as reported for *Pectobacterium* Phage vB_PatM_CB7, which was stable at 55 °C and survived at 60 °C, albeit with a significant drop in titer.

Since SSBI34 shares only 7% nucleotide homology with any of the associated phages in *Vequintavirinae*, there is not much to relate it to other phages in this subfamily. However, protein homology places it in the same subfamily but not among any existing genera. The protein homology (BLASTp) indicated that SSBI34 possesses 11tRNA molecules for 10 different amino acids, including two for selenocystine placement. This is in agreement with other members of the *Vequintavirinae* that possess a handsome pool of tRNA genes (between 1 and 18 tRNA genes), which may be involved in alternate codon usage [55]. Usually, the tRNAs retained in phages are those whose codons are highly used by phage genomes rather than the corresponding host [56]; however, a comparative study by Delesalle proposed that it may contribute more to amino acid usage than codon usage [55]. The fact that virulent phages contain more tRNA genes than temperate phages leads to higher codon usage bias. The presence of 11 tRNA may have contributed to the virulence of SSBI34. The presence of RNA polymerase (a smaller peptide of 153 AA) and tRNA may indicate that SSBI34 and similar phages (CR9, CR3, PBES02) possess higher fitness in comparison to the host [56].

A distinct system for DNA methylation to protect DNA from host enzymes and host restriction modification and restriction alleviation is found in SSBI34 and appears to be conserved across genera of *Vequintavirinae*; however, the restriction alleviation protein has less homology with other members (39% query cover). The proteome seems to have a mosaic arrangement, as different genes belong to different genera of *Vequintavirinae*. In the SSBI34 genome, enzymes involved in DNA replication and nucleotide metabolism were conserved across *Vequintavirinae*, as they were found in several members of the different genera with high homology. The majority of structural proteins involved in head formulation had high homology with *Certrevirus* phages; however, tail and baseplate assembly proteins (homology levels of less than 75%) showed a combination of unclassified *Certrevirus* division and unclassified *Vequintavirinae*. Tail fiber proteins had less homology with any member of the subfamily.

To warrant the use of a lytic phage as a biocontrol agent, a broad host range is desirable [57]; however, SSBI34 had a narrow host range, as it lysed two *S.* Typhimurium isolates out of 14 *Salmonella enterica* isolates tested and lysed no other member of *Enterobacteriaceae* tested. This is in agreement with the reports on CB7 and other members of *Certrevirus*, as they are known to have a narrow host range [53,54]. This narrow host range may result from tail fiber proteins interacting with a particular receptor on the host surface [58]. However, more experimental evidence is required. SSBI34 restricted the growth of the host strain to background levels for 16 h when observed by growth reduction assay in liquid cultures. Similar results were reported by Hyung for PBES02, where the phage restricted host growth until 10 h post-incubation. This indicates that SSBI34 may have potential as a therapeutic agent to curtail *S*. Typhimurium growth. However, the definitive therapeutic potential of SSBI34 can only be evaluated by in vivo analysis.

SSBI34 possesses a putative *Sle*B-type hydrolase for cell wall lysis. Other members of the *Certrevirus* genus had similar hydrolases in their genomes. However, the amino acid sequence of *Sle*B from SSBI34 shares only 50% similarity with any member of *Certrevirus,* indicating its unique nature, whereas cell wall hydrolases of CR3 and CR8 have maximum homology to each other (Figure 5).

## 5. Conclusions

*Salmonella*-phage-SSBI34 has a unique nucleotide sequence unlike any other in the NCBI database. The phage proteome is related to CR3, CR8, and CR9 phages of the *Certrevirus* genus in the subfamily *Vequintavirinae*; however, this was not enough to place SSBI34 in any existing genera. SSBI34 may have good therapeutic potential, as indicated by its ability to limit *S.* Typhimurium growth to background levels in liquid cultures, which can be further evaluated by in vivo experiments.

## Figures and Tables

**Table 1:** SSBI34 putative structural protein ORFs having BLASTp homology with various members of the subfamily *Vequintavirinae*.

S. No.	Position	ORF	Map Region	Total AA	Putative Function	Query Coverage	Similarity	GB Ac. No.	Organism
									
1	45,076–46,560	80	II	494 AA	Phage terminase, large subunit, PBSX family TC	99%	85.63%	YP_006383016.1	Cronobacter phage CR3
2	46,576–48,087	81	II	503 AA	Putative portal protein	99% 99%	77.51% 76.53%	YP_009014964.1 QQG33308.1	Cronobacter phage CR9 Pectobacterium phage PcCB7 V
3	48,156–48,733	82	II	191 AA	Putative prohead protease	98% 98%	77.37% 78.42%	QUL77265.1 YP_009014965.1	Escherichia phage UPEC06 Cronobacter phage CR9
4	49,814–50,248	84	II	144 AA	putative head stabilization/decoration protein	99%	65.36% 68.06% 66.67%	QEG12074.1 QUL77267.1 YP_009014967.1	Klebsiella phage vB_KaeM_KaOmega Escherichia phage UPEC06 Cronobacter phage CR9
5	50,272–51,264	85	II	330 AA	putative major capsid protein	99%	81.21%	YP_009014968.1 ATS9340	Cronobacter phage CR9 Pectobacterium phage DU_PP_I
6	51,998–55,276	89	II	1092 AA	Virion structural protein/ Putative tail fiber protein	99%	61.63% 60.54	ARB11484.1 YP_009014970.1	Pectobacterium phage vB_PatM_CB7 Cronobacter phage CR9
7	55,317–55,967	90	II	216 AA	Hypothetical protein (Putative structural protein tail fiber protein collagen triple helix)	99%	60.19% 53.46%	ATS9340 ARB11485.1	Pectobacterium phage DU_PP_I Pectobacterium phage vB_PatM_CB7
8	55,969–56,316	92	II	115 AA	Hypothetical protein putative tail fiber protein	92%	57.01%	YP_009014972.1	Cronobacter phage CR9
9	56,372–57,160	93	II	262 AA	hypothetical protein tail fiber like protein	91%	51.89%	YP_006383026.1	Cronobacter phage CR3
10	57,747–58,277	94	II	176 AA	hypothetical protein CR8 head completion adaptor	98%	72.57%	YP_009042249.1	Cronobacter phage CR8
11	58,340–58,801	95	II	153 AA	Putative RNA polymerase/virion morphogenesis protein	99%	75.82% 75.16%	ATS93412.1 YP_009014974.1	Pectobacterium phage DU_PP_I Cronobacter phage CR9
12	58,816–59,256	96	II	146 AA	hypothetical protein Putative minor capsid protein	99% 99%	75.34% 74.66%	YP_007392682.1 DAM41403.1	Pectobacterium phage phiTE Myoviridae sp.
13	59,256–59,834	97	II	192 AA	hypothetical protein putative tail to head joining protein	95% 90%	64.13% 66.68%	YP_006383030.1 QUL77277.1	Cronobacter phage CR3 Escherichia phage UPEC06
14	59,938–61,353	98	II	471 AA	Putative structural protein 1 (probable tail sheath protein)	99%	78.09% 77.87%	YP_006383031.1 YP_009042253.1	Cronobacter phage CR3 Cronobacter phage CR8
15	61,357–61,839	99	II	160 AA	Hypothetical protein putative tail tube protein	96%	76.28%	YP_009014978.1	Cronobacter phage CR9
16	61,916–62,389	100	II	157 AA	Tail assembly chaperon protein	96%	74.51%	YP_009042255.1	Cronobacter phage CR8
17	62,707–65,178	101	II	823 AA	Tail tape measure domain (controls tail length)	99% 84%	55.58% 60.96%	QEG12088.1 ATS93421.1	Klebsiella phage vB_KaeM_KaOmega Pectobacterium phage DU_PP_I
18	65,246–60,127	102	II	293 AA	Putative tail tape measure protein	98%	61.17%	QUL77284.1	Escherichia phage UPEC06
19	66,483–67,451	104	II	322 AA	Putative tail protein CR9	99%	73.62%	YP_009014984.1	Cronobacter phage CR9
20	67,461–68,108	105	II	215 AA	Putative base plate assembly protein	97%	66.23%	QEG12092.1	Klebsiella phage vB_KaeM_KaOmega
21	68,118–68,642	106	II	174 AA	Putative tail lysozyme/part of base plate wedge protein	99%	72.32% 71.19%	QEG12093.1 ARB11502.1	Klebsiella phage vB_KaeM_KaOmega Pectobacterium phage vB_PatM_CB7
22	68,737–70,224	107	II	495 AA	Putative base plate assembly protein	99%	76.97%	YP_009188994.1	Cronobacter phage PBES 02
23	70,236–70,883	108	II	215 AA	Putative base plate wedge protein	97% 97%	81.99% 78.20%	YP_009014988.1 QQG33332.1	Cronobacter phage CR9 Pectobacterium phage PcCB7V
24	70,897–72,417	109	II	506 AA	XXXCH domain containing protein (Putative tail fiber domain)	99%	50.90% 50.09%	QEG12096.1 YP_009042265.1	Klebsiella phage vB_KaeM_KaOmega Cronobacter phage CR8
25	72,420–72,935	110	II	171 AA	Putative tail fiber assembly protein	98% 98%	59.76% 57.65%	QUL77293.1 QEG12097.1	Escherichia phage UPEC06 Klebsiella phage vB_KaeM_KaOmega
26	73,313–73,774	112	II	153 AA	Putative membrane protein	99%	57.52% 55.56%	QEG12099.1 YP_009042268.1	Klebsiella phage vB_KaeM_KaOmega Cronobacter phage CR8
27	73,794–74,066	113	II	90 AA	Putative membrane protein	91%	73.49%	YP_009851591.1	Erwinia phage pEp_SNUABM_01
28	74,059–77,580	114	II	1173 AA	Putative tail fiber protein 2	99% 99%	32.98% 33.92%	QQG33338.1 YP_009189001.1	Pectobacterium phage PcCB7V Cronobacter phage PBES

**Table 2:** Comparison of genome characteristics of SSBI34 with other members of the subfamily *Vequintavirinae*.

Phage	Total ORF	Genome Size (bp)	G + C Content (%)	Accession No.	Querry Coverage	Shared Proteins (%) **
					Identity (%) *	
SE-Phage-SSBI34	234	141,095	44%	MZ520832	100	100%
					100	
Klebsiella phage vB_KaeM_KaOmega,	317	149,489	50.5	MN013077.1	7%	8.11%
					77.94%	
Cronobacter phage CR9	281	151,924	50.6	JQ691611.1	5%	17.52%
					74.78%	
Pectobacterium phage DU_PP_I	267	144,959	50.1	MF979560.1	5%	2.99%
					77.25%	
Pectobacterium phage PcCB7V	269	146,054	50.4%	MW367417.1	4%	6%
					77.78%	
Cronobacter phage PBES 02	270	149,732	50.7	KT353109.1	4%	5.55%
					76.56%	
Cronobacter phage CR8,	269	149,162 nt	50.8	KC954774.1	4%	7.69%
					76.44%	
Cronobacter phage CR3	265	149,273	50.9	JQ691612.1	4%	15%
					76.46%	
Pectobacterium phage DU_PP_IV	268	145,233	50.3	MF979563.1	3%	0
					76.49%	
Pectobacterium phage phiTE	242	142,349	50.1	JQ015307.1	2%	6.83%
					76.23%	
Acinetobacter phage ABPH49	252	149,960	50.8	MH533020.1	1%	0.85%
					72.87%	
Escherichia phage UPEC06	318	143,140	41.2	MW250786.1	1%	8.54%
					76.50%	

* Nucleotide sequence of SSBI34 compared by BLASTn. ** Protein similarity of each phage with SSBI34 (BLASTp).

## Data Availability

The genome sequence of *Salmonella*-phage-SSBI34 has been submitted to the NCBI Gene Bank and can be accessed by accession number MZ520832.

## Supplementary Materials

The following supporting information can be downloaded at: https://www.mdpi.com/article/10.3390/v14020241/s1, Table S1: Characterization of oligonucleotides used in *S.* *Typhimurium* strain SE-BS17. Table S2: The homology of SSBI34 ORFs with known proteins in the NCBI database (BLASTp).
