# Supplementary material for: Genome Analysis and Therapeutic Evaluation of a Novel Lytic Bacteriophage of Salmonella Typhimurium: Suggestive of a New Genus in the Subfamily Vequintavirinae
Source: Viruses. 2022 Jan 25;14(2):241. doi: 10.3390/v14020241 (PMC8879037; doi:10.3390/v14020241)
Supplement: Supplementary file 1 [file viruses-14-00241-s001.zip › viruses-1506079-supplementary.pdf]

Table S1. Oligonucleotides used in *Salmonella* Typhimurium strain SE-BS17 Characterization.

Primer code	Target Gene	Oligonucleotide Sequence (5´-3´)	Size (bp)
Salmonella identification	*invA*	F′ TCATCGCACCGTCAAAGGAACC<br>R′ GTGAAATTATCGCCACGTTCGG GGCAA	284
	iroB	F′ TGCGTATTCTGTTTGTCGGTCC<br>R′ TACGTTCCCACCATTCTTCCC	606
	fliC	F′ TATGCCGCTACATATGATGAG<br>R′ TTAACGCAGTAAAGAGAG	495
16S rRNA	PCR primers	27F 5' AGA GTT TGA TCM TGG CTC AG 3<br>1492R 5' TAC GGY TAC CTT GTT ACG ACT T 3'	Macrogen Korea
	Sequencing Primers	785F 5' GGA TTA GAT ACC CTG GTA 3'<br>907R 5' CCG TCA ATT CMT TTR AGT TT 3'	
Trimethoprim resistance gene	*dfrA1*	F′ GTGAAACTATCACTAATGG<br>R′ TTAACCCTTTTGCCAGATTT	474bp
Beta-Lactams	*blaCTX-M*	F′ CGCTGTTGTTAGGAAGTGTG<br>R′ GGCTGGGTGAAGTAAGTGAC	754
	blaTEM-1	F′ CAGCGGTAAGATCCTTGAGA<br>R′ ACTCCCCGTCGTGTAGATAA	643

Table S2. SSBI34 ORFs having BLAST P homology with known protein in NCBI data base.

Serial no	Position (bp)	Genome ORF	Map region	Total AA	Putative Function	Query coverage (BLAST P)	Similarity (BLAST P)	GB Ac. NO	Organism
1	1809-2141	5	I	110AA	Putative methanogen marker protein 2	98%	48.18%	CAD5242204.1	Klebsiella phage JD18
2	6123-6713	17	I	196AA	Putative serine tRNA ligase	91%	46.70 %	QUL77179.1	Escherichia phage UPEC06
3	7243-8358	19	I	371AA	Putative nucleotidyltransferase	98%	66.03%	YP_009015173.1	Cronobacter phage CR9
4	9986-11305	24	I	439AA	Putative tRNA nucleotidyltransferase	98%	67.97%	YP_009188904.1	Cronobacter phage PBES 02
5	12167-12682	28	I	171AA	phage N-6-adenine-methyltransferase	98%<br>98%	83.43%<br>81.76%	QQG33511.1<br>YP_006383219.1	Pectobacterium phage PcCB7V<br>Cronobacter phage CR3
6	12682-14028	29	I	448AA	ATP-dependent Clp endopeptidase, proteolytic subunit	55%	45.68%	YP_007392603.1	Pectobacterium phage phiTE
7	15673-15951	34	I	92AA	glutaredoxin 3, grxC	97%	52.69%	YP_006383227.1	Cronobacter phage CR3
8	16506-17603	37	I	365AA	ribonucleoside-diphosphate reductase, class 1b, (nrdF)	99%	76.16%	QUL77203.1	Escherichia phage UPEC06
9	17725-19989	38	I	754AA	ribonucleoside-diphosphate reductase, alpha subunit	99%	74.63%	YP_009042457.1	Cronobacter phage CR8
10	20828-21844	41	I	338AA	thymidylate synthase, flavin-dependent, thyX_41	99%<br>99%	85.25%<br>85.25%	YP_009188923.1<br>YP_009188923.1	Cronobacter phage CR8<br>Cronobacter phage PBES 02

11	22863-23510	44	I	215AA	ribulose-phosphate 3-epimerase, rpe_44	97%	65.71%	YP_009015200	Cronobacter phage CR9 .
12	23598-24644	45	I	348AA	DNA polymerase III, epsilon sub unit, dnaQ_45	99%	78.61%	YP_009188928.1<br>YP_009042465.1	Cronobacter phage PBES 02<br>Cronobacter phage CR8
13	24881-25441	47	I	186AA	Endo VII packaging and recombination endonuclease (present in T4 phages only lytic cycle no lysogeny)	95%	62.22%	YP_007392629.1	Pectobacterium phage phiTE
14	26878-27987	51	I	369AA	Putative exodeoxyribonuclease	99%	79.08%	QUL77220.1	Escherichia phage UPEC06
15	28335-28835	52	I	166AA	putative HNH endonuclease	99%	81.93%<br>81.33%	YP_009042472.1<br>YP_006383246.1	Cronobacter phage CR8<br>Cronobacter phage CR3
16	29635-30009	55	I	124AA	hypothetical protein CR9_253<br>Putative anti restriction endonuclease	96%<br>98%	73.55%<br>68.29%	YP_009015215.1<br>AYD80895.1	Cronobacter phage CR9<br>Klebsiella phage KP179
17	31215-32018	59	I	267AA	113 gène Product	91%	39.08%	AXN57777.1	Acinetobacter phage ABPH49
18	32482-33861	61	I	459AA	DNA ligase D, ligase domain, ligD_61	98%	71.65%	QUL77236.1	Escherichia phage UPEC06
19	34623-35012	62	I	129AA	putative transposase-like protein	71%	64.52%	QUL77240.1	Escherichia phage UPEC06
20	35015-35251	63	I	78AA	Queuosine biosynthesis protein QueD, queD_65	98%	58.97%	QEG12341.1	Klebsiella phage vB_KaeM_KaOmega
21	37233-38147	69	I	304AA	Ribose-phosphate di phosphokinase, prs_71	95%	49.15%	YP_009188960.1	Cronobacter phage PBES 02

22	38205-39995	70	I	596AA	Putative nictotinate phospho-ribosyl-transferase	99%	75.84%	YP_009188961.1<br>QEG12348.1	Cronobacter phage PBES 02<br>Klebsiella phage vB_KaeM_KaOmega
23	41529-41888	75	I	119AA	HNH endonuclease [Bacillus velezensis]	72%	59.77%	QCW19103.1	Salmonella phage 7t3
24	48730-49797	83	II	355AA	pyridine nucleotide-disulfide oxidoreductase	97%	64.62%<br>62.95%	YP_007392673.1<br>ARB11480.1	Pectobacterium phage phiTE<br>Pectobacterium phage vB_PatM_CB7
25	58340-58801	95	II	153AA	Putative RNA polymerase	99%	75.82%<br>75.16%	ATS93412.1<br>YP_009014974.1	Pectobacterium phage DU_PP_I<br>Cronobacter phage CR9
26	88022-90040	132	III	672AA	hypothetical protein<br>(Putative serine protease)	99%	65.38%<br>65.33%	YP_009015018.1<br>QQG33359.1	Cronobacter phage CR9<br>Pectobacterium phage PcCB7V
27	90351-90533	134	III	60AA	hypothetical protein<br>putative RNA triphosphatase	91%<br>57%	83.93%<br>82.86%	YP_006383073.1<br>QQG33364.1	Cronobacter phage CR3<br>Pectobacterium phage PcCB7V
28	93236-94078	139	III	280AA	19 gene product	99%	76.79%	YP_004893825.1	Salmonella phage PVPSE1
29	95585-98212	142	III	875AA	DNA polymerase I, polA_143	99%	82.37%	YP_006383075.1	Cronobacter phage CR3
30	99161-99529	146	III	122AA	Restriction alleviation protein, La	39%	51.02%	YP_007392476.1	Pectobacterium phage phiTE
31	102081-104078	154	III	665AA	phage replicative helicase, DnaB family_155	98%	82.13%	YP_009015046.1	Cronobacter phage CR9
32	104920-105174	159	III	84AA	Cell division topological specificity factor Mi	95%	48.15%	YP_009015051.1	Cronobacter phage CR9

33	105587-106957	162	III	456AA	Ti-type conjugative transfer relaxase TraA,<br>Tra (putative helicase)	99%<br>99%	70.61%<br>70.55%	YP_009189062.1<br>YP_009042324.1	Cronobacter phage PBES 02<br>Cronobacter phage CR8
34	107343-107864	165	III	173AA	spore cortex-lytic enzyme, sleB_166<br>Cell wall hydrolase	98%<br>98<br>95%	51.16%<br>51.15%<br>52.35%	YP_009187858.1<br>YP_006383102.1<br>QXL90429.1%	Klebsiella phage vB_KpnM_KB57<br>Cronobacter phage CR3<br>Salmonella phage NINP
35	107925-109334	166	III	469AA	(Role:166): RNA ligase, Rnl2 family_167	73%	50.43%	YP_009187854.1	Klebsiella phage vB_KpnM_KB57
36	109327-109833	167	III	168AA	Putative phosphatase putative hydrolase	98%<br>98%	64.46%<br>66.27%	YP_006383104.1<br>QEG12165.1	Cronobacter phage CR3<br>Klebsiella phage vB_KaeM_KaOmega
37	110701-111144	172	III	147AA	Phosphonate metabolism protein	97%	77.08%	YP_009015065.1	Cronobacter phage CR9
38	115193-116101	183	III	302AA	218 gene product	93%	38.33%	YP_004894025.1	Salmonella phage PVPSE1
39	116792-117349	185	III	185AA	RelA/SpoT family protein_186 putative metal dependent phosphohydrolase	95%<br>98%	66.85%<br>64.86%	QEG12186.1<br>YP_009015084.1	Klebsiella phage vB_KaeM_KaOmega<br>Cronobacter phage CR9
40	119198-120418	191	III	406AA	Hypothetical protein Ribosomal protein uS19, rpsS_192	98%	62.1%	YP_009015088.1	Cronobacter phage CR9
41	120492-122060	192	III	522AA	Hypothetical protein<br>Putative ATPase	98%<br>98%	62.13%<br>61.76%	YP_009015088.1<br>QEG12191.1	Cronobacter phage CR9<br>Klebsiella phage vB_KaeM_KaOmega

42	130673-131035	215	IV	120AA	SH3 domain protein_218	84%	52.43%	QHJ84486.1	Siphoviridae sp.
